# Vicarious Menstruation

**Published:** 1829-05

**Authors:** 


### VICARIOUS MENSTRUATION.

### *Case of Vicarious Menstruation, treated at the* Hôtel Dieu.

A WOMAN, nineteen years of age, had been severely burnt upon the arm, the skin of which was nearly totally destroyed. The menses had been previously suppressed; and, for five successive months, a sanguineous discharge took place from the wound upon the arm, at the usual periods of menstruation. By the application of leeches to the vagina, and by attention to the injured surface of the arm, the hemorrhage was checked. A periodical mucous discharge from the vagina followed, but, at the time this report was made, the natural discharge of the menses had not occurred.†

† Journal Hebdomadaire.

